# Systemic inflammatory response index over neutrophil–lymphocyte ratio and monocyte–lymphocyte ratio: comparison of prognostic performance in predicting major adverse cardiac events **[Regarding: Prognostic value of systemic inflammatory response index in patients with acute coronary syndrome undergoing percutaneous coronary intervention]**

**DOI:** 10.1080/07853890.2022.2104919

**Published:** 2022-08-02

**Authors:** Sridhar Mangalesh, Sharmila Dudani

**Affiliations:** a Department of Medicine, Army College of Medical Sciences, New Delhi, India; b Department of Pathology, Army College of Medical Sciences, New Delhi, India

To the Editor,

We read with keen interest the study by Han et al. [1]. The authors have comprehensively reported the role of a novel inflammatory marker, the systemic inflammatory response index (SIRI), in patients undergoing percutaneous coronary intervention (PCI) following an acute coronary syndrome (ACS). Their retrospective analysis demonstrated that the SIRI effectively predicted major adverse cardiac events (MACE) and improved the performance of the Global Registry of Acute Coronary Events (GRACE) score. Due to integrating both the neutrophil–lymphocyte ratio (NLR) and the monocyte–lymphocyte ratio (MLR) into one index, the SIRI should theoretically outperform both its components. The diagnostic and prognostic utility of the NLR and MLR has been confirmed previously in several disease conditions including cardiovascular diseases [2,3].

In a similar attempt at expanding the NLR, the systemic immune-inflammation index (SII), a composite of the NLR and the platelet-lymphocyte ratio (PLR) has been studied in many disease conditions. The SII has been shown to have a role in the prognosis of various malignancies, coronary artery disease, and ACS, among others [4,5]. Not unlike the SIRI, the SII is suggested to outperform the NLR and PLR due to the presence of the platelet count in the equation. The inclusion of the platelet count by the authors [1] in their analysis may have been a worthwhile addition to the study to determine if prognostic performance in predicting MACE improves even further with this change. Both SIRI and SII have a common NLR component, and studies robustly comparing the effectivity of either SIRI or SII with that of NLR alone are lacking.

The authors correctly state that the SIRI might be a more sensitive and useful biomarker than either NLR or MLR alone. Whether this is true is an important research question, and while the authors report the c-statistics individually for neutrophil, monocyte, and lymphocyte counts alone, those of the NLR and MLR are not reported. A formal comparison of the performance of NLR, MLR, and SIRI has not been conducted and would have provided valuable insight. Downstream adoption and acceptance of novel parameters by clinicians are of paramount importance in the final implementation of any risk stratification tool. More recently, the NLR has gained prominence as an excellent predictive marker in COVID-19 and is well-recognized and already widely used in clinical practice by physicians worldwide. Future studies are required to conclusively establish the superiority of novel biomarkers like the SIRI over existing parameters such as the NLR to allow for an evidence-based and widespread adoption of new markers into clinical practice and for the exploration of related therapeutic aspects.

## Data Availability

No original data has been utilized in this manuscript.
